# Association of uric acid in serum and urine with subclinical renal damage: Hanzhong Adolescent Hypertension Study

**DOI:** 10.1371/journal.pone.0224680

**Published:** 2019-11-15

**Authors:** Yang Wang, Chen Chen, Yu Yan, Yue Yuan, Ke-Ke Wang, Chao Chu, Jia-Wen Hu, Qiong Ma, Yue-Yuan Liao, Bo-Wen Fu, Ke Gao, Yue Sun, Yong-Bo Lv, Wen-Jing Zhu, Lei Yang, Jie Zhang, Rui-Hai Yang, Jun Yang, Jian-Jun Mu

**Affiliations:** 1 Department of Cardiovascular Medicine, First Affiliated Hospital of Xi’an Jiaotong University, Xi’an, China; 2 Key Laboratory of Molecular Cardiology of Shaanxi Province, Xi’an, China; 3 Department of Cardiology, Second Affiliated Hospital of Xi’an Jiaotong University, Xi’an, China; 4 Department of Cardiology, Xi’an Fourth People’s Hospital, Xi’an, China; 5 Institute of Cardiovascular Sciences, Hanzhong People’s Hospital, Hanzhong, China; Universita degli Studi di Perugia, ITALY

## Abstract

**Background and objectives:**

The aim of the study was to examine the associations of uric acid (UA) in blood and urine with subclinical renal damage (SRD) and its progression in a Chinese cohort.

**Methods:**

1) 2342 participants from our previously established cohort who were followed up in 2017 were included. Cross-sectional analysis was used to examine the relationships between serum and urinary UA and the risk of SRD. 2) A total of 266 participants were recruited from the same cohort in 2013, and followed up in 2017. Longitudinal analysis was used to determine the relationships of serum and urinary UA with progression of SRD, which was defined as urinary albumin-to-creatinine ratio (uACR) progression or estimated glomerular filtration rate (eGFR) decline.

**Results:**

In cross-sectional analysis, higher levels of uACR were associated with higher levels of serum uric acid (SUA) and urinary uric acid/creatinine ratio (uUA/Cre). Lower eGFR was associated with higher levels of SUA and fractional excretion of uric acid (FEUA) but lower uUA/Cre levels in all subjects. In addition, the multivariate-adjusted odds ratios for SRD compared with non-SRD were 3.574 (2.255–5.664) for uUA/Cre. Increasing uUA/Cre levels were associated with higher risk of SRD. In longitudinal analysis, 4-year changes of uUA/Cre and SUA were significantly associated with eGFR decline.

**Conclusions:**

This study suggested that urinary UA excretion was significantly associated with the risk of SRD in Chinese adults. Furthermore, 4-year changes of serum and urinary UA were associated with SRD progression. These findings suggest that UA, especially urinary UA, may be used as a simple, noninvasive marker for early detection of decreased renal function in otherwise healthy subjects.

## Introduction

Chronic kidney disease (CKD) has become a major public health issue because of the global prevalence and the associated increase in the incidence of cardiovascular disease and premature death [1,2]. Studies have reported that the prevalence of CKD among adults is 13.0% in United States and 10.8% in China [3,4]. Subjects with early-stage CKD always have no symptoms, and the majority in the early stage of CKD remains undiagnosed even in developed countries [1]. In addition, CKD, from the earliest stages, is associated with a high risk of cardiovascular events [2]. Therefore, identifying the markers of early renal damage and risk factors associated with its progression are useful for establishing effective therapeutic strategies to prevent the onset and progression of end-stage renal disease and the accompanying cardiovascular complications.

Uric acid (UA) is the final oxidation product of purine catabolism in humans [5,6]. For decades, it has been hypothesized that the antioxidant properties of UA might be protective against oxidative stress, oxidative cell injury and ageing [7]. However, recent clinical and cohort studies suggest that hyperuricaemia is a risk factor for cardiovascular events and renal disease [8,9]. An elevated albuminuria and impaired glomerular filtration rate (GFR) are important indicators for grading the severity of renal damage and predicting long-term prognosis. Several studies have shown the relationships of serum uric acid (SUA) with albuminuria and decreased GFR, which has been observed in subjects with diabetes mellitus, hypertension and heart failure [8,10–12]. Recently, few studies have indicated that hyperuricaemia is associated with subclinical renal damage (SRD), defined as slightly increased albuminuria or decreased estimated GFR (eGFR), in hypertensive patients [13,14]. However, such an association is still unclear in the general population, especially for people who are non-hypertensive and non-diabetic.

Although multiple studies have shown that hyperuricaemia is common in CKD patients [8,15,16], the association between urinary UA excretion and target organ damage is rarely studied. UA homeostasis is under tight regulation, with the kidney assuming a pivotal role. Approximately two-thirds of UA in blood is easily filtered by glomeruli into the renal tubule [17]. Nearly 90% of filtered UA is reabsorbed by the S1 segment of the proximal convoluted tubule, and the remaining 10% of them is finally excreted into urine [17]. Previous studies showed that elevated urinary UA was a suspected risk factor for calcium oxalate kidney stones in subjects with calcium nephrolithiasis [18,19]. Recently, studies indicate that the urinary UA can be used as a simple, noninvasive parameter of the severity of disease and mortality. For instance, urinary uric acid/creatinine ratio (uUA/Cre) has found to be remarkably higher in hypoxic premature infants or in neonates with birth asphyxia, and this ratio was correlated significantly with the clinical severity of the disease [20–22]. However, the relationship between urinary UA and early renal damage has not been published previously. In addition, there is virtually no data on urinary UA in relation with the progression of SRD.

In the present study, we therefore conducted cross-sectional analysis based on our previously established cohort to investigate the relationships of UA in blood and urine with SRD. Furthermore, in the longitudinal analysis, we also aimed to determine the relationship between UA and the progression of SRD, which was defined as uACR progression or eGFR decline during 4-year follow-up.

## Materials and methods

### Study cohort

The study population was derived from the Hanzhong Adolescent Hypertension Study, which was established in 1987. This ongoing prospective, population-based cohort study of 4623 adolescents who regularly undergo several follow-ups for investigating the development of cardiovascular risk factors originating from children and young adults (S1 Fig). Details of the study protocol have been published elsewhere [23,24].

#### This study was divided into two sections

**Fig 1:**
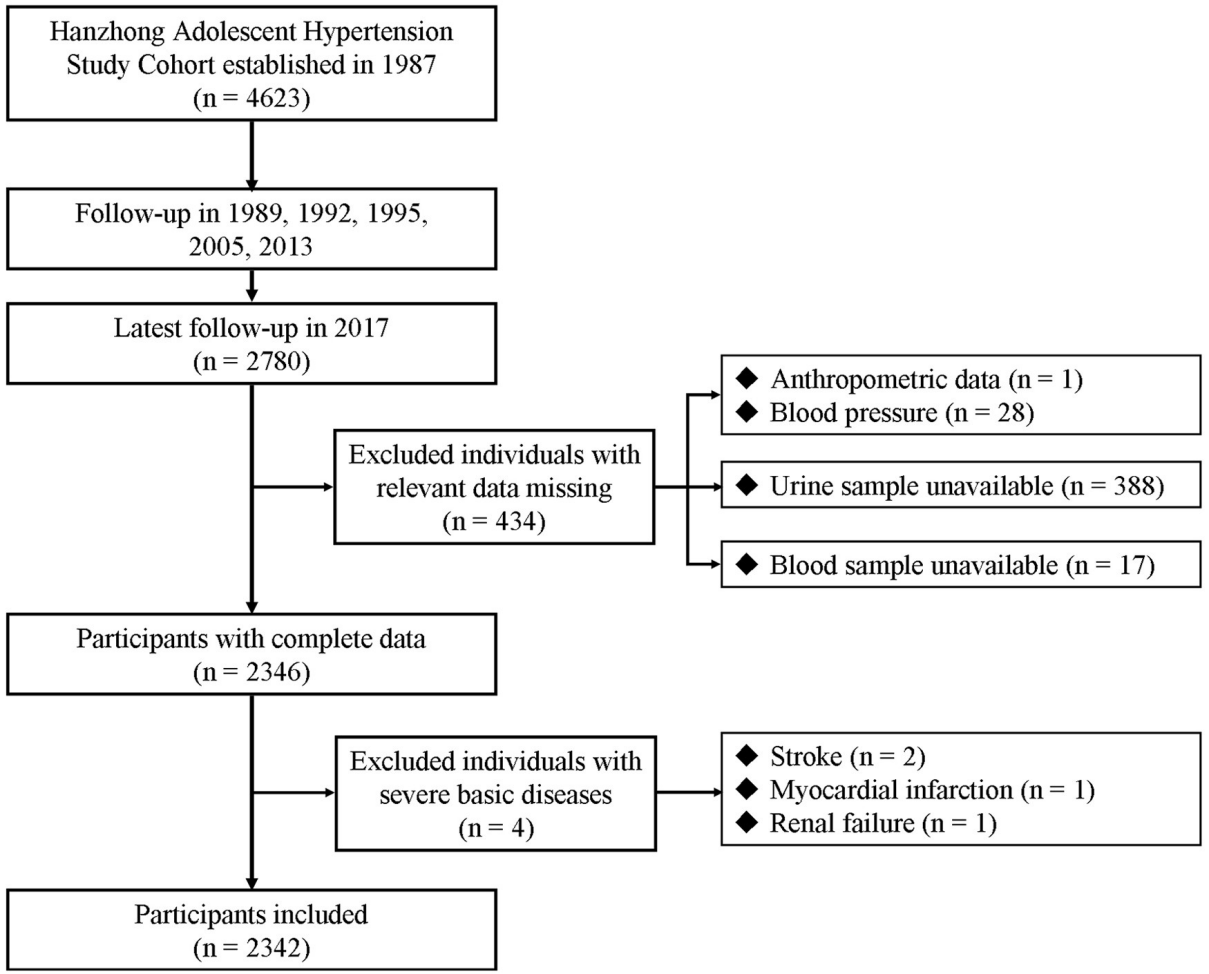
Flow diagram for recruitment of participants in cross-sectional study.

1. In order to explore the associations of UA in blood and urine with SRD, we used cross-sectional analysis of data from the large cohort that was followed up in 2017. The participant selection process is described in Fig 1. Participants were excluded if they had missing data on serum or urinary biochemistry (N = 17 and 388, respectively), blood pressure (N = 28), height and weight (N = 1) and if they had a self-identified history of coronary heart disease, renal failure or stroke (N = 4), leaving 2342 subjects for our primary analyses. The outcome variable was the risk of SRD, and the potential exposure variables for clinical outcome were the increased levels of SUA and urinary UA excretions in the cross-sectional analysis.
2. To further examine the relationships of changes in serum and urinary UA with SRD progression, a 4-year longitudinal analysis was also conducted in this study. We used data from a small cohort of 338 subjects that was created based on the large cohort in 2005. The detailed study design and procedures have been published previously [25,26]. We followed up with this small cohort in 2013 and 2017. For the current analysis, we did not measure the serum and urinary levels of UA and creatinine in 2005, so the small cohort that was followed up in 2013 was considered the baseline for investigating the association of changes in serum and urinary UA with the progression of SRD (2013–2017). Participants who were lost to follow-up in 2017 (N = 70) and those with missing data on anthropometry and blood pressure (N = 2) were excluded. The remaining 266 subjects were included in the analysis. No significant difference was observed when these subjects were excluded from the cohort (S1 Table). The potential exposure variables were 4-year change in serum and urinary UA, and the outcome variables was the progression of SRD, which was defined as the uACR progression or eGFR decline.

Written informed consent was obtained from each subject, and the study protocol was approved by the Ethics Committee of the First Affiliated Hospital of Xi’an Jiaotong University (code: 2015–128). This study was performed in accordance with the Declaration of Helsinki (2008). (ClinicalTrials.gov. registration number: NCT02734472).

### Anthropometric measurements

Participants completed standardized questionnaires that inquired about past medical history, current medications, alcohol and tobacco use, family history and physical activity. Body weight and height were measured. Body mass index (BMI) was calculated as weight (kg) divided by height (m^2^). Blood pressure (BP) was measured in the sitting position using a standard mercury sphygmomanometer as previously described [23,24,27,28]. Hypertension was defined as a systolic BP of ≥ 140 mm Hg, a diastolic BP ≥ 90 mm Hg or as the use of antihypertensive drugs according to subjects’ clinical data or self-report.

### Blood biochemical analyses

Venous blood samples were obtained, immediately centrifuged, and then stored at –80 °C until analysis. Standardized measurements for lipid profile [triglycerides, total cholesterol, low-density lipoprotein (LDL), high-density lipoprotein (HDL)], serum uric acid (SUA), serum creatinine, alanine aminotransferase (ALT), aspartate transaminase (AST) and fasting glucose were measured by an automatic biochemical analyser (model 7600; Hitachi, Ltd., Tokyo, Japan). Hyperuricemia was defined as SUA level of ≥ 420μmol/L in men or ≥ 360μmol/L in women. eGFR was calculated using the formula adapted from the Modification of Diet in Renal Disease equation on the basis of data from Chinese subjects with CKD [23,24,29].

### Urinary biochemical testing

The first-void and mid-stream urine was collected, followed by venous blood sampling. Urinary levels of creatinine, albumin and UA were measured by an automatic biochemical analyser (Hitachi, Ltd., Japan). Details of these assays were described previously [23,27,28]. The urinary albumin-to-creatinine ratio (uACR) was calculated by dividing urinary albumin in mg by urinary creatinine in mmol (mg/mmol). UA is transported in the proximal tubule by secretory and reabsorbing transporters, and its handling is a useful marker of proximal tubular function [30,31]. Fractional excretion of uric acid (FEUA) quantifies the percent of filtered UA that is excreted into urine. FEUA was calculated from the standard formula: ([urinary uric acid]×[serum creatinine])/([urine creatinine]×[serum uric acid])×100, expressed as percentage. The presence of subclinical renal damage (SRD) was defined as an uACR of ≥ 3.5 mg/mmol in women and 2.5 mg/mmol in men or an eGFR between 30 and 60 ml/min/1.73 m^2^, as previously reported [14,24,32].

### Statistical analysis

Data are expressed as the means ± standard deviations for normally distributed values, as geometric mean (interquartile range) for non-normally distributed values, and as percentages. The differences between the groups were calculated using the χ^2^-test, Student’s t-test and Mann–Whitney test as appropriate. Analysis of variance (ANOVA) was used to test the linearity across quintiles. We first performed linear and logistic regression analyses to test associations of SRD with UA in serum and urine (cross-sectional analyses). The dependent variables were the risk of SRD while the potential confounders were age, gender, hypertension, diabetes, BMI, total cholesterol and triglycerides. We next performed linear regression analyses to investigate relationships of changes in serum and urinary UA with SRD progression (longitudinal analyses). uACR progression or eGFR decline was used as dependent variable, and 4-year changes of serum and urinary UA were also used as covariates in the models. All variables were checked for multicollinearity before multivariate analysis, and the variables with multicollinearity were excluded. All statistical analyses were conducted using SPSS 16.0 (SPSS, Inc., Chicago, IL). Statistical significance is set as a 2-sides *P* value of <0.05.

## Results

### Characteristics of participants in a cross-sectional study

Table 1 presents the characteristics of subjects according to the quartiles of uUA/Cre levels. Participants with higher UA excretions tended to have higher HDL, eGFR, FEUA and uACR levels but lower SUA, ALT, AST, LDL, serum creatinine, and a lower prevalence of hyperuricemia, smoking and alcohol use.

**Table 1:** Baseline characteristics according to uUA/Cre levels in all subjects (n = 2342).

	Quartiles of uUA/Cre				
Characteristics	I (< 0.12)	II (0.12–0.20)	III (0.20–0.33)	IV (> 0.33)	*P* for trend
No. of subjects	587	585	587	583	–
Age (years)	43.0 (40.0–45.0)	43.0 (40.0–45.0)	43.0 (40.0–45.0)	43.0 (40.0–45.0)	0.289
Alcohol consumption (n, %)	192 (32.7)	197 (33.7)	163 (27.8)	131 (22.5)	<0.001
Current smoking (n, %)	306 (52.1)	276 (47.2)	250 (42.6)	176 (30.2)	<0.001
Diabetes mellitus (n, %)	21 (3.6)	25 (4.3)	35 (6.0)	16 (2.7)	0.832
Hypertension (n, %)	129 (22)	117 (20)	123 (25.7)	110 (23.0)	0.592
Hyperuricemia (n, %)	43 (7.3)	39 (6.7)	34 (5.8)	23 (3.9)	0.012
BMI (kg/m^2^)	24.1±3.2	24.1±3.1	24.0±3.1	24.0±3.1	0.368
Heart rate (beats/min)	69.0 (63.5–75.5)	68.5(63.5–75.0)	69.5 (63.0–76.0)	69.0 (63.0–76.0)	0.833
SBP (mmHg)	121.3 (112.7–132.0)	121.3 (113.3–130.3)	121.3 (112.0–130.3)	121.3 (112.0–131.7)	0.995
DBP (mmHg)	76.3 (69.7–84.3)	76.0 (70.0–84.0)	75.3 (68.0–83.3)	75.7 (68.3–83.0)	0.870
SUA (μmol/L)	293.6 (242.8–353.7)	287.1 (230.0–340.1)	275.2 (226.3–330.2)	256.6 (211.5–313.2)	<0.001
Fasting glucose (mmol/L)	4.52 (4.22–4.89)	4.58 (4.31–4.94)	4.59 (4.29–4.93)	4.58 (4.30–4.90)	0.202
ALT (U/L)	20.0 (14.0–30.0)	19.0 (14.0–27.0)	19.0 (13.0–27.0)	17.0 (12.0–23.0)	<0.001
AST (U/L)	17.0 (13.0–21.0)	16.0 (13.0–20.0)	16.0 (13.0–20.0)	15.0 (13.0–20.0)	0.003
Total cholesterol (mmol/L)	4.57 (4.10–5.14)	4.52 (4.03–5.01)	4.47 (4.00–4.94)	4.48 (4.07–4.99)	0.171
Triglycerides (mmol/L)	1.37 (0.97–2.01)	1.36 (0.99–1.96)	1.32 (0.97–1.91)	1.25 (0.90–1.81)	0.125
LDL (mmol/L)	2.55 (2.15–2.99)	2.52 (2.14–2.92)	2.44 (2.08–2.85)	2.48 (2.14–2.88)	0.008
HDL (mmol/L)	1.15 (0.99–1.32)	1.10 (0.98–1.31)	1.15 (0.99–1.33)	1.18 (1.01–1.38)	<0.001
Serum creatinine (μmol/L)	80.4±14.7	77.9±13.3	76.2±13.7	72.6±14.2	<0.001
eGFR (mL/min/1.73m^2^)	94.0 (84.4–105.6)	96.5 (86.9–108.1)	98.2 (87.6–111.0)	100.7 (89.1–115.1)	<0.001
uUA/Cre	0.08 (0.06–0.09)	0.16 (0.13–0.18)	0.25 (0.22–0.28)	0.46 (0.38–0.62)	<0.001
FEUA	2.08 (1.43–2.63)	4.22 (3.48–5.06)	6.74 (5.64–8.26)	13.4 (10.4–18.8)	<0.001
uACR (mg/mmol)	0.73 (0.49–1.24)	0.88 (0.62–1.42)	1.02 (0.72–1.70)	1.42 (0.86–2.49)	<0.001

BMI, body mass index; SBP, systolic blood pressure; DBP, diastolic blood pressure; SUA, serum uric acid; ALT, alanine aminotransferase; AST, aspartate transaminase; LDL, low-density lipoprotein; HDL, high-density lipoprotein; uUA/Cre, urinary uric acid/creatinine ratio; FEUA, fraction excretion of uric acid; uACR, urinary albumin-to-creatinine ratio; Non-normally distributed variables are expressed as the median (interquartile range). All other values are expressed as mean ± SD or n, %.

### Associations of serum and urinary UA levels with uACR and eGFR

uACR levels were positively correlated with sex, the prevalence of hypertension and diabetes, total cholesterol, SUA (*β* = 0.093, *P*<0.001), and uUA/Cre (*β* = 0.042, *P* = 0.041) but inversely correlated with age. In addition, eGFR was negatively related to age, total cholesterol, SUA (*β* = -0.300, *P*<0.001) but positively related to uUA/Cre levels (*β* = 0.086, *P*<0.001) in all subjects (Table 2). Furthermore, uUA/Cre [3.574 (2.255–5.664), *P*<0.001] was significantly associated with the risk of SRD after adjusting for multiple confounders. However, SUA [1.000 (0.998–1.002), *P* = 0.676] and FEUA [1.009 (0.999–1.019), *P* = 0.080] were not associated with SRD (Table 3).

**Table 2:** Relationship between various characteristics and uACR and eGFR (n = 2342).

Characteristics	uACR		eGFR	
	*β*	*P* value	*β*	*P* value
Gender (Male)	0.050	0.019	0.041	0.054
Age (years)	-0.047	0.021	-0.073	<0.001
Hypertension (%)	0.106	<0.001	-0.031	0.146
Diabetes mellitus (%)	0.050	0.016	0.037	0.074
BMI (kg/m^2^)	0.034	0.121	-0.016	0.467
Total cholesterol (mmol/L)	0.112	<0.001	-0.109	<0.001
Triglycerides (mmol/L)	0.024	0.278	-0.018	0.426
SUA (μmol/L)	0.093	<0.001	-0.300	<0.001
uUA/Cre	0.042	0.041	0.086	<0.001
FEUA	0.012	0.549	-0.019	0.368

eGFR, estimated glomerular filtration rate; uACR, urinary albumin-to-creatinine ratio; BMI, body mass index; SUA, serum uric acid; uUA/Cre, urinary uric acid/creatinine ratio; FEUA, fraction excretion of uric acid. The variables of smoking status, alcohol consumption, SBP, DBP, fasting glucose, serum creatinine, LDL, HDL and heart rate were excluded due to multicollinearity.

**Table 3:** Association between various characteristics and the presence of SRD (n = 2342).

Characteristics	Odds Ratios (confidence interval)	*P* value
Gender (Male)	1.199 (0.911–1.576)	0.195
Age (years)	0.979 (0.942–1.017)	0.268
Hypertension (%)	3.506 (2.650–4.638)	<0.001
Diabetes mellitus (%)	3.889 (2.415–6.263)	<0.001
BMI (kg/m^2^)	1.087 (1.042–1.133)	<0.001
Total cholesterol (mmol/L)	1.078 (0.920–1.265)	0.353
Triglycerides (mmol/L)	1.136 (1.039–1.242)	0.005
SUA (μmol/L)	1.000 (0.998–1.002)	0.676
FEUA	1.009 (0.999–1.019)	0.080
uUA/Cre	3.574 (2.255–5.664)	<0.001

Logistic regression analyses were used to test the risk of SRD, after adjustment for age, gender, hypertension, diabetes, BMI, total cholesterol and triglycerides. The variables of smoking status, alcohol consumption, SBP, DBP, fasting glucose, serum creatinine, LDL, HDL and heart rate were excluded because of multicollinearity. SRD, subclinical renal damage; BMI, body mass index; SUA, serum uric acid; FEUA, fraction excretion of uric acid; uUA/Cre, urinary uric acid/creatinine ratio.

We further assessed the effect of UA excretion on the risk of SRD when uUA/Cre was modelled in quartiles (Table 4). In an age- and sex-adjusted model, the ORs (95% CIs) of SRD across increasing quartiles of uUA/Cre were 1.00, 1.115 (0.767–1.622), 1.427 (0.996–2.045) and 2.303 (1.632–3.251) (*P* for trend < 0.001). In the multivariate model, further adjusting for hypertension, diabetes, BMI, total cholesterol and triglycerides, the ORs (95% CIs) were 1.00, 1.122 (0.756–1.664), 1.362 (0.928–1.998) and 2.480 (1.719–3.578), respectively; linear trend *P* < 0.001.

**Table 4:** Association between each quartile of uUA/Cre and presence of SRD (n = 2342).

			Odds Ratios (95% confidence interval)	
	Non-SRD controls	SRD patients	Age, sex-adjusted	Multivariate*
Quartile 1	528 (26.0%)	59 (18.7%)	1.00 (reference)	1.00 (reference)
Quartile 2	521 (25.1%)	64 (20.3%)	1.115 (0.767–1.622)	1.122 (0.756–1.664)
Quartile 3	508 (25.1%)	79 (25.1%)	1.427 (0.996–2.045)	1.362 (0.928–1.998)
Quartile 4	470 (23.2%)	113 (35.9%)	2.303 (1.632–3.251)	2.480 (1.719–3.578)
*P* for trend	<0.001	<0.001	<0.001	<0.001

*Logistic regression analyses were used to test the risk of SRD, after adjustment for age, gender, hypertension, diabetes, BMI, total cholesterol and triglycerides. The variables of smoking status, alcohol consumption, SBP, DBP, fasting glucose, serum creatinine, LDL, HDL and heart rate were excluded due to multicollinearity. SRD, subclinical renal damage; FEUA, fraction excretion of uric acid; uUA/Cre, urinary uric acid/creatinine ratio.

### Participant characteristics in the longitudinal study

The characteristics of those who participated in both surveys are presented in Table 5. On average, subjects had higher uUA/Cre, FEUA, uACR, serum creatinine, heart rate, LDL and total cholesterol; lower eGFR, DBP, HDL and SUA levels; and a higher proportion of alcohol consumption after a 4-year follow-up.

**Table 5:** Characteristics of the study participants at baseline and follow-up (n = 266).

Characteristics	Baseline in 2013	Follow-up in 2017	*P* value
Gender (M/F)	152/114	152/114	–
Age (years)	37.0 (35.0–40.0)	41.0 (39.0–44.0)	<0.001
Current smoking (n, %)	113 (42.5)	120 (45.1)	0.541
Alcohol consumption (n, %)	26 (9.8)	78 (29.3)	<0.001
Hypertension (n, %)	84 (31.6)	65 (24.4)	0.067
Diabetes mellitus (n, %)	5 (1.9)	16 (6.0)	0.014
BMI (kg/m^2^)	24.6±3.7	24.7±3.5	0.695
Heart rate (beats/min)	66.0(60.0–72.0)	70.0(63.5–76.5)	<0.001
SBP (mmHg)	122.0(114.0–132.0)	122.7(114.3–133.3)	0.505
DBP (mmHg)	81.0(74.0–90.0)	77.2(70.3–85.8)	<0.001
Fasting glucose (mmol/L)	4.49 (4.26–4.71)	4.61 (4.29–4.93)	0.002
Total cholesterol (mmol/L)	4.34±0.79	4.58±0.79	0.001
Triglycerides (mmol/L)	1.42(1.00–2.13)	1.38(0.98–2.00)	0.452
LDL (mmol/L)	2.36(2.00–2.74)	2.51(2.11–2.90)	0.010
HDL (mmol/L)	1.65±0.32	1.17±0.26	<0.001
SUA (μmol/L)	316.3±87.7	295.2±81.2	0.004
uUA/Cre	0.08(0.04–0.15)	2.30(1.92–2.84)	<0.001
FEUA	1.92(1.00–3.69)	4.71(2.74–7.81)	<0.001
Serum creatinine (μmol/L)	76.6±14.4	81.0±12.8	<0.001
eGFR (ml/min/1.73 m^2^)	99.1(87.2–112.9)	89.4(77.5–108.3)	<0.001
uACR (mg/mmol)	0.33(0.25–0.43)	1.05(0.65–2.02)	<0.001

BMI, body mass index; SBP, systolic blood pressure; DBP, diastolic blood pressure; LDL, low-density lipoprotein; HDL, high-density lipoprotein; SUA, serum uric acid; eGFR, estimated glomerular filtration rate; uACR, urinary albumin-to-creatinine ratio. uUA/Cre, urinary uric acid/creatinine ratio; FEUA, fraction excretion of uric acid. Non-normally distributed variables are expressed as the median (interquartile range). All other values are expressed as mean ± SD or n, %.

Men had lower eGFR during the two follow-ups than women, and men had a higher rate of eGFR decline (-4.3±4.0 *vs*. 1.0±6.1 mL/min/1.73m^2^/y, *P* < 0.001). However, uACR progression rate was not significantly different between men and women (Table 6).

**Table 6:** Change of the uACR and eGFR over time.

	Male subjects	Female subjects	*P*-value
No. of sujects	152	114	–
uACR data (mg/mmol)			
uACR 2013,	0.59 (0.40–0.98)	0.85 (0.53–1.50)	0.002
uACR 2017	1.00 (0.62–1.87)	1.23 (0.70–2.18)	0.207
uACR progression	0.38 (-1.24–0.96)	0.33 (-1.64–1.10)	0.533
uACR progression rate, per year	0.10 (-0.31–0.24)	0.08 (-0.41–0.28)	0.533
eGFR data (mL/min/1.73m^2^)			
eGFR 2013	99.3±17.4	105.1±22.9	0.019
eGFR 2017	80.2 (73.2–89.8)	108.7 (90.9–125.2)	<0.001
eGFR decline	-17.3±16.0	4.0±24.3	<0.001
eGFR decline rate, per year	-4.3±4.0	1.0±6.1	<0.001

uACR, urinary albumin-to-creatinine ratio; eGFR, estimated glomerular filtration rate; Non-normally distributed variables are expressed as the median (interquartile range). All other values are expressed as mean ± SD.

### Changes in serum and urinary UA levels for SRD progression

The relationships of 4-year changes in serum and urinary UA levels with SRD progression (2013–2017) are shown in Table 7. The 4-year changes in uUA/Cre (*β* = -0.114, *P* = 0.040) and SUA (*β* = 0.187, *P* = 0.001) were significantly associated with eGFR decline. However, we did not find an association between changes in UA and uACR progression over a 4-year follow-up (Table 7).

**Table 7:** Associations of 4-year changes in serum and urinary UA with the progression of SRD.

	uACR progression		eGFR decline	
	*β*	*P* value	*β*	*P* value
4-year change				
SUA (μmol/L)	-0.009	0.890	0.187	0.001
uUA/Cre(μmol/L)	0.016	0.796	-0.114	0.040
FEUA(μmol/L)	0.005	0.941	-0.103	0.065

Included in the multivariate regression models: age, gender, hypertension, diabetes, BMI, total cholesterol, triglycerides and eGFR at baseline. The variables of smoking status, alcohol consumption, SBP, DBP, fasting glucose, serum creatinine, LDL, HDL and heart rate were excluded due to multicollinearity. SRD, subclinical renal damage; UA, uric acid; eGFR, estimated glomerular filtration rate; uACR, urinary albumin-to-creatinine ratio; SUA, serum uric acid; FEUA, fraction excretion of uric acid; uUA/Cre, urinary uric acid/creatinine ratio.

### Sensitivity analysis

Several sensitivity analyses were performed. Firstly, we removed subjects with hyperuricemia under treatment in the cross-sectional study (n = 11) and longitudinal trial (n = 3) to exclude the potential influence of urate-lowering drugs. As shown in S2–S5 Tables, all the results remained similar after adjustment for potential confounders. In addition, to further exclude the potential influence of antihypertensive or hypoglycemic medications, we performed all analyses by excluding individuals with diabetes, hypertension or hyperuricemia under treatment in the cross-sectional study (n = 92) and longitudinal trial (n = 26), and similar results were obtained (S6–S9 Tables).

## Discussion

To the best of our knowledge, the present study is the first to evaluate the relationship between urinary UA excretion and the risk of SRD. Interestingly, we showed that a higher uUA/Cre category was significantly associated with an increased OR for the presence of SRD compared with the reference group. The observed positive association between uUA/Cre and SRD consistently occurred when uUA/Cre was considered a continuous variable. These data suggest that the urinary UA may act as a simple noninvasive, cost-effective, single biochemical marker for assessing the severity of early renal damage.

UA homeostasis is determined by the dynamic balance between its production and excretion, and the latter mainly includes intestinal and renal pathway [33]. In“renal excretion” pathway, the renal proximal tubule is an important regulator and the primary site of urate re-absorption [30] and is responsible for almost all the renal urate transportation in the kidney [33]. Several studies indicate that urinary UA has the potential to act as a simple, noninvasive indicator for evaluating the disease development and mortality. Chen et al. [21] showed that the uUA/Cre was significantly increased in hypoxic premature infants compared with in hypoxic term ones. Bahubali et al. [22] reported that uUA/Cre was higher in neonates with birth asphyxia, and this ratio was closely associated with the severity of disease. Nariman et al. [20] found that uUA/Cre levels increased with the clinical severity of disease and was correlated with adverse outcome and longer duration of stay in neonatal intensive care unit (NICU). Subclinical renal damage (SRD), defined as slightly elevated albuminuria and decreased GFR, was significantly associated with the increased incidence of CKD and associated cardiovascular complications. Few studies support the possible role of SUA as a risk factor for SRD in hypertensive subjects. Viazzi et al. [13] showed that hypertensive patients with increased SUA levels showed early signs of renal damage. The authors further showed that for each SD increment in SUA, there was a 69% increased risk of developing microalbuminuria and a 39% increased risk of ultrasound-detectable abnormalities [13]. Mulèet et al. suggested that the presence of SRD was significantly associated with SUA in patients with hypertension [14]. However, the association of urinary UA excretion with the risk of SRD has not been fully understood. In the present study, we firstly showed that uUA/Cre was significantly associated with the presence of SRD. In fact, UA per se can be detrimental to the kidneys, as shown in recent studies. Verzola et al. [34] demonstrated that UA internalized by urate transporter 1 (URAT1) could promote reactive oxygen species (ROS)-induced tubular cell apoptosis by upregulating Nox4 expression at tubular level. They further showed that UA could trigger oxidative and inflammatory response mediated by toll-like receptor 4 (TLR4). In addition, cotreatment with UA and angiotensin II (Ang II) exert an additive unfavorable cellular effect, which cannot be completely prevented by renin-angiotensin-aldosterone system (RAAS) inhibitors [35]. These studies suggest the complex interaction between damage-associated molecular patterns (DAMPs), such as UA and Ang II, innate immunity and the development of renal damage.

To our knowledge, this study is the first report concerning the association between urinary UA and the progression of SRD. We found that urinary UA change over 4 years was associated with eGFR decline in this Chinese population. Previous studies suggested that elevated SUA levels were significantly associated with GFR decline. For example, Akasaka et al. [36] attempted to examine the impact of SUA on the natural history of eGFR. They showed that elevation of SUA accelerated eGFR decline, and it was a result instead of a cause. De Cosmo et al. [37] found that SUA levels were significantly correlated with eGFR decline in a large cohort of type 2 diabetes (T2D) patients. Moreover, Wang et al. [11] conducted a prospective cohort study and found that elevated SUA was positively associated with incident eGFR decline. They further showed that for every 100 μmol/L increase in SUA, there was a 33% increased risk of incident eGFR decline [11]. Xu et al. [12] recently reported an association between increased SUA and greater GFR reduction during a 5-year follow-up in patients with T2D. In keeping with these observations, in our study, we found that SUA levels were negatively associated with eGFR in our cross-sectional study and that the change in SUA during a 4-year period was positively associated with eGFR decline. Our findings derived from cross-sectional and longitudinal cohort studies further confirmed the negative correlation between SUA and eGFR decline in the general population. Based on these previous findings, the present study further identified a significant association between urinary UA excretions and eGFR decline. Future studies investigating mechanisms underlying this phenomenon can be of interesting.

Albuminuria not only an important prognostic indicator for renal damage, and but also a marker of endothelial dysfunction [38]. Increased albuminuria was associated with cardiovascular risk for kidney damage and vascular injury [39,40]. Previously, several studies found that SUA was positively associated with albuminuria and as a useful predictor for the development of albuminuria, including in subjects with hypertension [16] and diabetes mellitus [8]. Uric acid-lowering drugs have been found to decrease proteinuria in T2D patients [41]. In addition, Li et al. [42] recently reported that uACR was significantly correlated with 24-h urinary excretion of UA and its clearance rate, but not with FEUA, after adjusting for potential confounders in CKD patients. Consistent with this result, our study demonstrates that uACR levels were correlated with SUA and uUA/Cre, not with FEUA, in Chinese adults in the general population. Furthermore, Scheven et al. showed [10] that albuminuria was positively associated with tubular UA reabsorption. This relationship was demonstrated by Zou et al. from another perspective in that urinary UA excretion was significantly decreased in proteinuric patients than in healthy individuals [43]. Future large prospective trials are still needed to verify the relationship between urinary UA excretion and the progression of albuminuria.

Some limitations of our study deserve mention. Firstly, our results are obtained from northern Chinese individuals and consequently cannot be directly extrapolated to other ethnic groups. Secondly, we use spot urine specimens to determine urinary albumin excretion in present study. Nevertheless, 24-h urine is difficult to collect, and spot urine samples are more practical for use in large-scale studies. uACR has been verified to closely associate with estimates of microalbuminuria in 24-h urine and is effective in predicting kidney diseases [44,45]. Thirdly, all subjects in this study were youth and middle aged between 36 and 45 years during the follow-up at 2017, and thus the findings may not be generalizable to other age groups. Finally, some participants were lost during the follow-up. However, baseline characteristics in 2013 were similar between the participants and non-participants, and the study cohort seems to be representative of the original study population.

In summary, our study shows that urinary UA excretion is significantly associated with the risk of early renal damage in Chinese adults. However, we failed to find a significant relationship between serum UA and SRD. Our results also indicate that 4-year changes of UA in blood and urine are associated with eGFR decline in this Chinese population. These findings suggest that UA, especially urinary UA, may be used as a simple, noninvasive marker for early detection of decreased renal function in otherwise healthy subjects. Further clinical trials targeting hyperuricosuria can be of much interest.

## Supporting information

S1 File Minimal data set. (XLS)

S1 Fig The protocol of longitudinal follow-up of the cohort. (TIF)

S1 Table Comparison of baseline characteristic between those who did and did not participate in the longitudinal study. (DOC)

S2 Table Relationship between various characteristics and uACR and eGFR in subjects without urate-lowering treatment. (DOC)

S3 Table Association between various characteristics and the risk of SRD in subjects without urate-lowering treatment. (DOC)

S4 Table Association between each quartile of uUA/Cre and presence of SRD in subjects without urate-lowering treatment. (DOC)

S5 Table Associations of 4-year changes in serum and urinary UA with the progression of SRD in subjects without urate-lowering treatment. (DOC)

S6 Table Relationship between various characteristics and uACR and eGFR in subjects without medication use. (DOC)

S7 Table Association between various characteristics and the risk of SRD in subjects without medication use. (DOC)

S8 Table Association between each quartile of uUA/Cre and presence of SRD in subjects without medication use. (DOC)

S9 Table Associations of 4-year changes in serum and urinary UA with the progression of SRD in subjects without medication use. (DOC)
