# Supplementary figures and images for: Association of uric acid in serum and urine with subclinical renal damage: Hanzhong Adolescent Hypertension Study
Source: PLoS One. 2019 Nov 15;14(11):e0224680. doi: 10.1371/journal.pone.0224680 (PMC6857911; doi:10.1371/journal.pone.0224680)

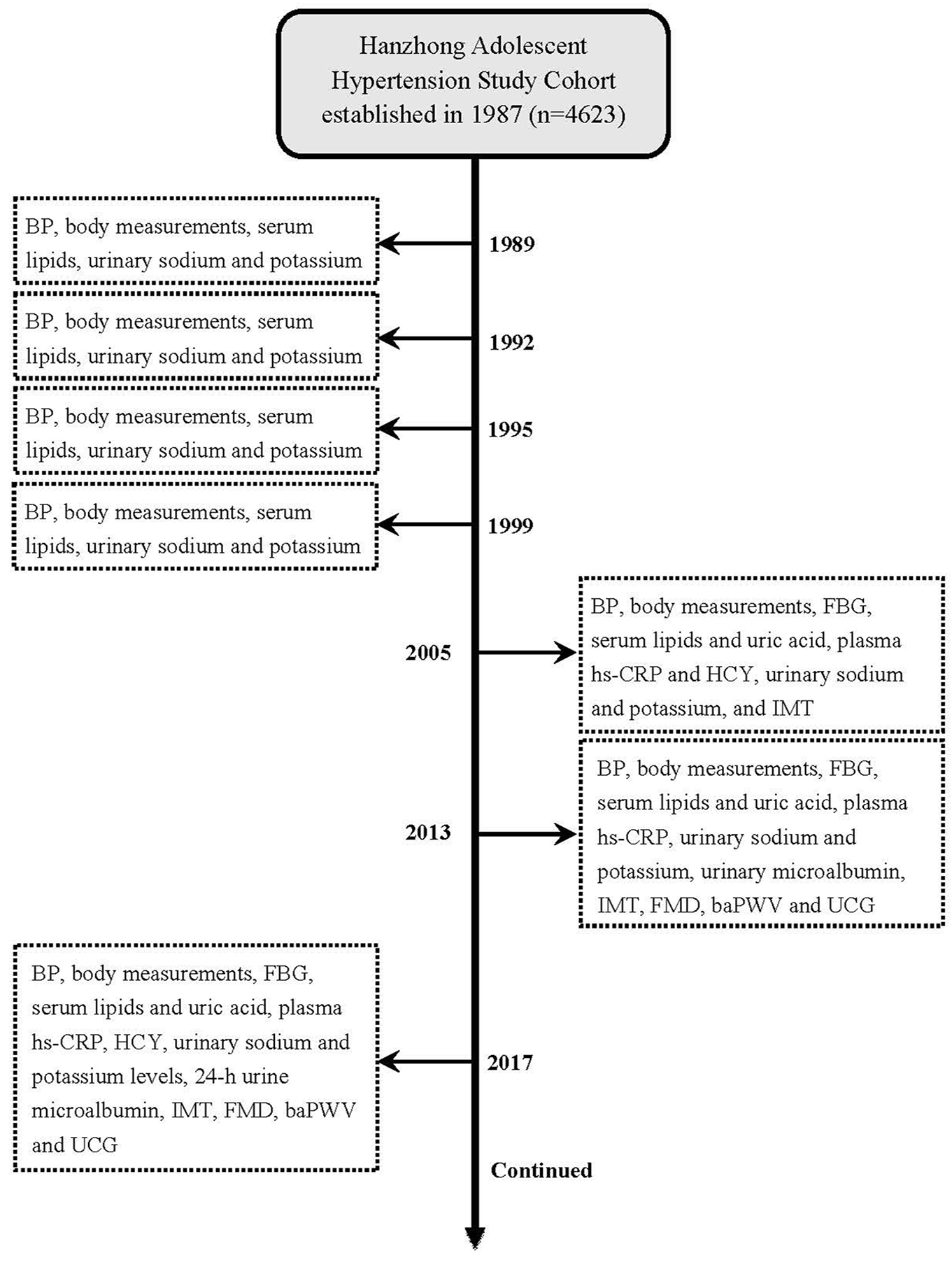

Supplement: S1 Fig — (TIF) [file pone.0224680.s002.tif]
