# Supplementary material for: Association of uric acid in serum and urine with subclinical renal damage: Hanzhong Adolescent Hypertension Study
Source: PLoS One. 2019 Nov 15;14(11):e0224680. doi: 10.1371/journal.pone.0224680 (PMC6857911; doi:10.1371/journal.pone.0224680)
Supplement: S1 Table — (DOC) [file pone.0224680.s003.doc]

**S1 Table.** Comparison of baseline characteristic between those who did and did not participate in the longitudinal study

| **Characteristics** | **Participants** | **Nonparticipants*** | ***P*-value** |
| --- | --- | --- | --- |
| No. of subjects | 266 | 72 | − |
| Age (years) | 37.0(35.0-40.0) | 36.0(34.0-40.0) | 0.420 |
| BMI (kg/m^2^) | 24.6±3.7 | 23.8±3.9 | 0.083 |
| Heart rate (beats/min) | 66.4±9.4 | 68.2±11.1 | 0.114 |
| SBP (mmHg) | 122.0(114.0-132.0) | 122.0(112.0-130.0) | 0.713 |
| DBP (mmHg) | 81.0(74.0-90.0) | 80.0(72.0-90.0) | 0.520 |
| Fasting glucose (mmol/L) | 4.5(4.3-4.7) | 4.5(4.2-4.8) | 0.514 |
| Total cholesterol (mmol/L) | 4.3±0.8 | 4.3±0.7 | 0.677 |
| Triglycerides (mmol/L) | 1.4(1.0-2.1) | 1.3(1.0-1.9) | 0.249 |
| LDL (mmol/L) | 2.4±0.6 | 2.4±0.6 | 0585 |
| HDL (mmol/L) | 1.7(1.4-1.9) | 1.7(1.4-1.9) | 0.717 |
| CRP (μmol/L) | 0.6(0.3-1.1) | 0.6(0.3-1.1) | 0.598 |
| Homocysteine (μmol/L) | 9.0(6.8-11.7) | 10.0(7.6-13.9) | 0.053 |
| Serum creatinine (μmol/L) | 76.1(67.6-85.4) | 80.3(70.2-89.2) | 0.013 |
| SUA (μmol/L） | 316.3±87.7 | 307.1±86.5 | 0.354 |
| uACR (mg/mmol) | 0.69(0.45-1.3) | 0.82(0.51-1.32) | 0.125 |
| eGFR (ml/(min·1.73m^2^)) | 99.1(87.2-112.9) | 92.2(81.5-102.5) | <0.001 |

*Nonparticipants (*N*=72), including subjects lost to follow-up in 2017 (*N*=70), or with missing data on blood pressure and anthropometry (*N*=2). BMI, body mass index; SBP, systolic blood pressure; DBP, diastolic blood pressure; LDL, low-density lipoprotein; HDL, high-density lipoprotein; CRP, C-reactive protein; SUA, serum uric acid; uACR, urinary albumin to creatinine ratio; eGFR, estimated glomerular filtration rate. Non-normally distributed variables are expressed as the median (interquartile range). All other values are expressed as mean ± SD or n, %.
