# Supplementary material for: Association of uric acid in serum and urine with subclinical renal damage: Hanzhong Adolescent Hypertension Study
Source: PLoS One. 2019 Nov 15;14(11):e0224680. doi: 10.1371/journal.pone.0224680 (PMC6857911; doi:10.1371/journal.pone.0224680)
Supplement: S2 Table — (DOC) [file pone.0224680.s004.doc]

**S2 Table.** Relationship between various characteristics and uACR and eGFR in subjects without urate-lowering treatment (n=2331)

| **Characteristics** | **uACR** | | **eGFR** | |
| --- | --- | --- | --- | --- |
|  | ***β*** | ***P* value** | ***β*** | ***P* value** |
| Gender | 0.050 | 0.019 | 0.041 | 0.058 |
| Age (years) | -0.047 | 0.023 | -0.072 | <0.001 |
| Hypertension (%) | 0.107 | <0.001 | -0.029 | 0.182 |
| Diabetes mellitus (%) | 0.053 | 0.011 | 0.042 | 0.045 |
| BMI (kg/m^2^) | 0.036 | 0.107 | -0.014 | 0.523 |
| Total cholesterol (mmol/L) | 0.111 | <0.001 | -0.110 | <0.001 |
| Triglycerides (mmol/L) | 0.024 | 0.290 | -0.020 | 0.377 |
| SUA (μmol/L) | 0.094 | <0.001 | -0.299 | <0.001 |
| uUA/Cre | 0.042 | 0.043 | 0.086 | <0.001 |
| FEUA | 0.013 | 0.535 | -0.018 | 0.372 |

eGFR, estimated Glomerular Filtration Rate; uACR, urinary albumin-to-creatinine ratio; BMI, body mass index; SUA, serum uric acid; uUA/Cre, urinary uric acid/creatnine ratio; FEUA, fraction of uric acid. The variables of smoking status, alcohol consumption, SBP, DBP, fasting glucose, serum creatinine, LDL, HDL and heart rate were excluded due to multicollinearity.
