# Supplementary material for: Association of uric acid in serum and urine with subclinical renal damage: Hanzhong Adolescent Hypertension Study
Source: PLoS One. 2019 Nov 15;14(11):e0224680. doi: 10.1371/journal.pone.0224680 (PMC6857911; doi:10.1371/journal.pone.0224680)
Supplement: S3 Table — (DOC) [file pone.0224680.s005.doc]

**S3 Table.** Association between various characteristics and the risk of SRD in subjects without urate-lowering treatment (n=2331).

| **Characteristics** | **Odds Ratios (confidence interval)** | ***P* value** |
| --- | --- | --- |
| Gender (Male) | 1.215 (0.923-1.601) | 0.165 |
| Age (years) | 0.978 (0.941-1.016) | 0.247 |
| Hypertension (%) | 3.648 (2.752-4.835) | <0.001 |
| Diabetes mellitus (%) | 4.280 (2.636-6.951) | <0.001 |
| BMI (kg/m^2^) | 1.087 (1.042-1.134) | <0.001 |
| Total cholesterol (mmol/L) | 1.074 (0.915-1.261) | 0.382 |
| Triglycerides (mmol/L) | 1.136 (1.039-1.242) | 0.005 |
| SUA (μmol/L) | 1.001 (0.998-1.003) | 0.588 |
| uUA/Cre | 3.641 (2.295-5.775) | <0.001 |
| FEUA | 1.009 (0.999-1.018) | 0.080 |

Logistic regression analyses were used to test the risk of SRD, after adjustment for age, gender, hypertension, diabetes, BMI, total cholesterol and triglycerides. The variables of smoking status, alcohol consumption, SBP, DBP, fasting glucose, serum creatinine, LDL, HDL and heart rate were excluded because of multicollinearity. SRD, subclinical renal damage; BMI, body mass index; SUA, serum uric acid; FEUA, fraction excretion of uric acid; uUA/Cre, urinary uric acid/creatinine ratio
