# Supplementary material for: Association of uric acid in serum and urine with subclinical renal damage: Hanzhong Adolescent Hypertension Study
Source: PLoS One. 2019 Nov 15;14(11):e0224680. doi: 10.1371/journal.pone.0224680 (PMC6857911; doi:10.1371/journal.pone.0224680)
Supplement: S5 Table — (DOC) [file pone.0224680.s007.doc]

**S5 Table.** Associations of 4-year changes in serum and urinary UA with the progression of SRD in subjects without urate-lowering treatment (n = 263).

|  | **uACR progression** | | **eGFR decline** | |
| --- | --- | --- | --- | --- |
|  | ***β*** | ***P* value** | ***β*** | ***P* value** |
| 4-year change |  |  |  |  |
| SUA (μmol/L) | -0.013 | 0.842 | 0.184 | 0.001 |
| uUA/Cre | 0.016 | 0.798 | -0.112 | 0.042 |
| FEUA | 0.005 | 0.940 | -0.100 | 0.071 |

Included in the multivariate regression models: age, gender, hypertension, diabetes, BMI, total cholesterol, triglycerides and eGFR at baseline. The variables of smoking status, alcohol consumption, SBP, DBP, fasting glucose, serum creatinine, LDL, HDL and heart rate were excluded due to multicollinearity. SRD, subclinical renal damage; UA, uric acid; eGFR, estimated glomerular filtration rate; uACR, urinary albumin-to-creatinine ratio; SUA, serum uric acid; FEUA, fraction excretion of uric acid; uUA/Cre, urinary uric acid/creatinine ratio.
