# Supplementary material for: Association of uric acid in serum and urine with subclinical renal damage: Hanzhong Adolescent Hypertension Study
Source: PLoS One. 2019 Nov 15;14(11):e0224680. doi: 10.1371/journal.pone.0224680 (PMC6857911; doi:10.1371/journal.pone.0224680)
Supplement: S6 Table — (DOC) [file pone.0224680.s008.doc]

**S6 Table.** Relationship between various characteristics and uACR and eGFR in subjects without medication use (n=2250).

| **Characteristics** | **uACR** | | **eGFR** | |
| --- | --- | --- | --- | --- |
|  | ***β*** | ***P* value** | ***β*** | ***P* value** |
| Gender | 0.050 | 0.023 | 0.034 | 0.116 |
| Age (years) | -0.035 | 0.092 | -0.067 | 0.001 |
| Hypertension (%) | 0.098 | <0.001 | -0.025 | 0.247 |
| Diabetes mellitus (%) | 0.034 | 0.103 | 0.044 | 0.035 |
| BMI (kg/m^2^) | 0.036 | 0.105 | -0.018 | 0.433 |
| Total cholesterol (mmol/L) | 0.114 | <0.001 | -0.111 | <0.001 |
| Triglycerides (mmol/L) | 0.016 | 0.480 | -0.020 | 0.370 |
| SUA (μmol/L) | 0.094 | 0.001 | -0.298 | <0.001 |
| uUA/Cre | 0.045 | 0.035 | 0.085 | <0.001 |
| FEUA | 0.013 | 0.547 | -0.019 | 0.377 |

eGFR, estimated Glomerular Filtration Rate; uACR, urinary albumin-to-creatinine ratio; BMI, body mass index; SUA, serum uric acid; uUA/Cre, urinary uric acid/creatnine ratio; FEUA, fraction of uric acid. The variables of smoking status, alcohol consumption, SBP, DBP, fasting glucose, serum creatinine, LDL, HDL and heart rate were excluded due to multicollinearity.
