# Supplementary material for: Association of uric acid in serum and urine with subclinical renal damage: Hanzhong Adolescent Hypertension Study
Source: PLoS One. 2019 Nov 15;14(11):e0224680. doi: 10.1371/journal.pone.0224680 (PMC6857911; doi:10.1371/journal.pone.0224680)
Supplement: S8 Table — (DOC) [file pone.0224680.s010.doc]

**S8 Table.**  Association between each quartile of uUA/Cre and presence of SRD in subjects without medication use (n = 2250)

|  |  |  | **Odds Ratios (95% confidence interval)** | |
| --- | --- | --- | --- | --- |
|  | **Non-SRD controls** | **SRD patients** | **Age, sex-adjusted** | **Multivariate*** |
| Quartile 1 | 512 (26.0%) | 51 (18.2%) | 1.00 (reference) | 1.00 (reference) |
| Quartile 2 | 505 (25.6%) | 58 (20.7%) | 1.164 (0.783-1.730) | 1.138 (0.752-1.722) |
| Quartile 3 | 494 (25.1%) | 68 (24.3%) | 1.407 (0.958-2.056) | 1.390 (0.929-2.079) |
| Quartile 4 | 459 (23.3%) | 103 (36.8%) | 2.365 (1.642-3.405) | 2.512 (1.711-3.688) |
| *P* for trend | <0.001 | <0.001 | <0.001 | <0.001 |

*****Logistic regression analyses were used to test the risk of SRD, after adjustment for age, gender, hypertension, diabetes, BMI, total cholesterol and triglycerides. The variables of smoking status, alcohol consumption, SBP, DBP, fasting glucose, serum creatinine, LDL, HDL and heart rate were excluded due to multicollinearity. SRD, subclinical renal damage; uUA/Cre, urinary uric acid/creatinine ratio.
